# Neutralization of Interleukin 1-beta is associated with preservation of thalamic capillaries after experimental traumatic brain injury

**DOI:** 10.3389/fneur.2024.1378203

**Published:** 2024-04-26

**Authors:** Ilknur Özen, Fredrik Clausen, Johanna Flygt, Niklas Marklund, Gesine Paul

**Affiliations:** ^1^Lund Brain Injury Laboratory for Neurosurgical Research, Department of Clinical Sciences, Lund University, Lund, Sweden; ^2^Department of Clinical Sciences Lund, Neurosurgery, Lund University, Skåne University Hospital, Lund, Sweden; ^3^Translational Neurology Group, Department of Clinical Science, Wallenberg Neuroscience Center and Wallenberg Center for Molecular Medicine, Lund University, Lund, Sweden; ^4^Department of Neurology, Scania University Hospital, Lund, Sweden

**Keywords:** traumatic brain injury, interleukin-1β, cytokine, pericytes, platelet derived growth factor (PDGFRβ), capillaries

## Abstract

**Introduction:**

Traumatic brain injury to thalamo-cortical pathways is associated with posttraumatic morbidity. Diffuse mechanical forces to white matter tracts and deep grey matter regions induce an inflammatory response and vascular damage resulting in progressive neurodegeneration. Pro-inflammatory cytokines, including interleukin-1β (IL-1β), may contribute to the link between inflammation and the injured capillary network after TBI. This study investigates whether IL-1β is a key contributor to capillary alterations and changes in pericyte coverage in the thalamus and cortex after TBI.

**Methods:**

Animals were subjected to central fluid percussion injury (cFPI), a model of TBI causing widespread axonal and vascular pathology, or sham injury and randomized to receive a neutralizing anti-IL-1β or a control, anti-cyclosporin A antibody, at 30 min post-injury. Capillary length and pericyte coverage of cortex and thalamus were analyzed by immunohistochemistry at 2- and 7-days post-injury.

**Results and Conclusion:**

Our results show that early post-injury attenuation of IL-1β dependent inflammatory signaling prevents capillary damage by increasing pericyte coverage in the thalamus.

## Introduction

Traumatic brain injury (TBI) is a heterogeneous disease with complex pathological mechanisms that consist of primary and secondary brain injury. The primary brain injury is caused by a mechanical force transmitted to the brain that can disrupt network function and results in a long-lasting secondary injury in connected remote areas (1). The process of secondary injury includes neuroinflammation, vascular and white matter changes, and blood brain barrier (BBB) dysfunction (2–4). Diffuse injury to white matter tracts and the vasculature in deep grey matter regions such as the thalamus, a region particularly vulnerable to TBI in both humans and rodents (5–7) contributes to post-traumatic morbidity.

The inflammatory response in the brain after diffuse TBI has been previously reported, which could be closely linked to a variety of pro-inflammatory cytokines (8, 9). Among them, Interleukin-1 beta (IL-1β) is a major pro-inflammatory cytokine produced in the brain by activated resident microglia, astrocytes, and endothelial cells (10–12) that acts through binding to its cell surface receptor IL-IR (12–14). Both moderate and severe TBI leads to increased levels of IL-1β mRNA and protein in the cortex and deep central brain structures, observed as early as 1 h post-injury (15, 16). This rapid production of IL-1β is involved in neuroinflammatory processes associated with neurodegeneration (11, 17).

Cerebral autoregulation (CA) maintains a consistent and adequate blood flow to the brain when the cerebral perfusion pressure (CPP) fluctuates (18). Adequate cerebral perfusion and oxygen delivery are crucial for early stages of TBI as reductions in cerebral blood flow (CBF) may lead to secondary pathologies and poor clinical outcome (19). Upon TBI, the release of inflammatory factors and the activation of immune cells can affect the cerebral vasculature (9, 20, 21). Consequently, the vascular inflammatory pathways could be a putative mechanism compromising the CBF, ultimately resulting in dysfunction of cerebral autoregulation (CA) (22). A few experimental studies using fluid percussion models demonstrated that acute post traumatic hypoperfusion is associated with disrupted microvascular integrity and decreased capillary density (23–25). Most importantly, a functional capillary network depends on dynamic changes in pericyte distribution and morphology coordinated across the capillary network (26). Given that pericytes are highly susceptible to brain injury and ischemia, any disruption of the crosstalk between pericyte and the vascular wall of capillaries (27, 28) can lead to pericyte malfunction and capillary degeneration during the subacute phase of brain injury (29, 30). Cerebrovascular injury is another common observation closely linked to inflammation in experimental TBI models (3, 25, 31). Since IL-1 receptors are expressed in both endothelial cells and pericytes, it is likely that the cerebrovasculature is a key target for inflammatory signals, including IL-1β (32). However, possible effects of IL-1β on the regional capillary network along with the pericyte response after TBI remain unclear.

The behavior analyses from our previous TBI studies in rodents indicate that concomitant changes in white matter structural integrity accompanied the alterations in functional connectivity between thalamocortical regions (20, 33, 34). Indeed, diffuse TBI can lead to motor deficits in rodents due to brain network dysfunction and damage to the thalamus, which gradually leads to neurodegeneration over time (35, 36). It has been suggested that post-injury inflammation may play a significant role as a secondary injury mechanism, both for the vasculature and for deep brain structures (9, 20, 37). IL-1β neutralization can reduce brain tissue loss and improve visuospatial learning and memory associated with thalamocortical regions in mouse models of brain injury (20). However, the underlying mechanisms by which IL-1β affects brain injury in these regions and vascular dysfunction are not yet fully understood. Therefore, we further hypothesized that post-injury inflammation may be a key secondary injury mechanism both for the vasculature and for deep brain structures such as the thalamus. To test this hypothesis, we used the central fluid percussion (cFPI) model in mice and investigated whether neutralizing IL-1β attenuates capillary damage by modulating pericyte morphology and number in the cortex and thalamus.

## Materials and methods

### Surgical procedure

This study utilizes tissue samples from a prior study with other objectives (33, 37) in line with the guideline principles at Karolinska Institute, with the aim to improve the ethical use of animals in testing according to the 3R principle. The total number of included animals in the study was 58 out of total 116 mice.

In the two-days post injury (dpi) group, the number of included animals per group was sham+CsA (*n* = 3), sham+IL-1β (*n* = 3–4), cFPI+CsA (*n* = 5), and cFPI+IL-1β (*n* = 4–5). In the seven-dpi group, the number of included animals per group was sham CsA (*n* = 3), sham+IL-1β (*n* = 3–4), cFPI+CsA (*n* = 4), and cFPI+IL-1β (*n* = 5). In the current study, we have analyzed the brains at 2 and 7 dpi, which were strategically selected based on previous findings and the functional outcomes of interest (20, 33, 35, 37).

Adult male mice C57BL/6 mice (pre-injury weight 25 ± 1.7 gram; Taconic, Denmark), were housed with free access to food and water for a minimum of 7 days prior to surgery. Seventy-eight C57BL/6 mice were subjected to cFPI as described previously (33, 37). Briefly, after making a craniotomy over the midline midway between the bregma and lambda sutures, a plastic cap was placed on it. The dura mater was kept intact. The saline cap filled with isotonic saline was attached to the Luer-Lock on the fluid percussion device (VCU Biomedical Engineering Facility, Richmond, VA). TBI was performed releasing the fluid percussion pendulum to strike a saline-filled cylinder in order to create a pressure wave transmitted into the closed cranial cavity. Thirty-eight sham-injured animals were subjected to an identical procedure as the cFPI animals except that the pendulum was not released.

### IL-1β neutralizing antibody administration

The mouse-specific IL-1β neutralizing antibody (01BSUR, 300 μg/dose) or as control, anti-cyclosporin A mlgG2a (CsA-Ab; 500 μg/dose) (kindly provided by Novartis Pharma AG, Basel, Switzerland) were administered intraperitoneally (ip) 30 min after sham injury or cFPI for all evaluated groups (33, 37). The antibody solutions were mixed with normal saline to obtain a 75 μg/mL concentration. Each mouse was administered 0.15–0.2 mL of the diluted solution, resulting in a dosage of 500 μg/kg.

### Tissue preparation

The mice were sacrificed at 2 and 7 dpi and transcardially perfused using 4% formaldehyde (HistoLab Products AB, Gothenburg). The brains were postfixed overnight at 4°C and placed in 30% sucrose solution. Brains were cut at 20 μm thickness, and all sections were stored in cryoprotectant buffer [30% glycerin, 30% ethylene glycol, and 40% 1x phosphate buffered saline (PBS)] at −20°C.

### Immunohistochemistry

Free-floating brain coronal sections were washed three times in phosphate buffer saline (PBS), brain sections were then blocked in 5% normal donkey serum (NDS) in PBS supplemented with 0.25% Triton X-100 (PBS-T) for 1 h. Primary antibodies were incubated overnight at room temperature or at 4°C in 3% NDS PBS-T. The following primary antibodies were used: for labeling pericytes anti- PDGFRβ (rabbit, 1:100, Cell Signaling, Danvers, United States), for blood vessel staining anti-podocalyxin (PODXL) (goat; 1:400, R&D Systems, Minneapolis, United States). For PDGFRβ detection, sections were subjected to heat-induced antigen retrieval in citrate buffer for 30 min at 80°C.

For brightfield staining of PDGFRβ, brain sections were quenched with a peroxidase solution (3% H_2_O_2_, diluted in PBS) for 15 min prior to blocking. After incubation with the primary antibody, sections were incubated with the corresponding anti-rabbit biotinylated secondary antibody (1:500, 711-065-152, Jackson Immunoresearch, Baltimore, United States), in 3% NDS PBS-T, at room temperature for 2 h, and the signal was enhancement by using Vectorstain ABC Elite kit (Vector Laboratories, CA, United States). Staining was revealed using chromogen 3,3-diaminobenzidine-tetrahydrochloride (Dabsafe, Saveen Werner AB, Limhamn, Sweden) and 3% H_2_O_2_. Sections were dehydrated in consecutive higher concentrations of ethanol, followed by xylene and mounted using Pertex (Histolab AB, Gothenburg, Sweden).

For immunofluorescence, after incubation with primary antibodies, sections were washed in PBS and incubated with fluorophore-conjugated secondary antibodies for double labeling of PDGFRβ and PODXL The secondary antibodies were used: CY2-conjugated donkey anti-rabbit (1:500, 711.225-152, Immunoresearch, Baltimore, United States) for PDGFRβ and Daylight 649-conjugated donkey anti-goat (1:500, 705-495-147, Immunoresearch, Baltimore, United States) for PODXL.

### Brightfield image processing and pericyte quantification

Sections stained with 3,3′-Diaminobenzidine (DAB) were analyzed using Olympus BX51 light microscope and cells counted using CellSens digital imaging software. Figures were composed using Photoshop CS5 software. To quantify the number of DAB-stained PDGFRβ^+^ pericytes, cells were identified by their typical pericyte morphology and counted manually in both the somatosensory cortex and the thalamus using CellSens digital imaging software (from AP −1.06 according to Bregma). Four optical fields of 0.5mm^2^ from each brain region were analysed at 20×. Three coronal sections, including the thalamus, were analyzed between −2.30 and −2.56 mm from bregma for each mouse.

### Pericyte coverage and capillary length

Pericyte capillary coverage was determined as the percentage of PDGFRβ^+^ pericytes surface area covering total PODXL^+^ capillary surface area per field (ROI) (224 × 224 μm) in the somatosensory cortex and thalamus. A maximum projection of 15 micrometer z-stacks was acquired from both the somatosensory cortex and thalamus section (from −1.06 according to Bregma) using a Zeiss LMS510 confocal microscope. A coronal section, including thalamus, was analyzed − 2.30 mm from bregma for each mouse. Four images of the somatosensory cortex and two images for the thalamus were obtained at 40X. The areas of PDGFRβ^+^ pericytes and PODXL^+^ blood vessels from capillaries defined by ≤10 μm diameter were separately subjected to threshold processing and the respective signal for each image was calculated using the NIH Image J Area Measurement tool.

For measurement of the total capillary length, the maximal projection of images at 40× magnification (Zeiss LMS510) was obtained for each section and capillary length assessed using the NIH Image J software, NeuroJ plugin tool (38). Briefly, PODXL-positive capillaries were subjected to threshold processing and a binary picture was created. Each capillary was outlined manually with the freehand line tool allowing to capture varying vascular shapes as previously described (38).

### Statistics

Graphs and statistical analysis were made with Prism v.8 (GraphPad Software). Since the study utilizes tissue samples from a prior study with other objectives, power analyses were initially performed according to those previous studies to estimate the effect of neutralizing IL-1β antibody. However, we had previously observed an approximately >60% decrease in the pericyte coverage from cFPI in preliminary experiments, which suggested a group size of *n* = 4 to be sufficient to detect the hypothesized difference when applied to the current study.

Shapiro–Wilk normality test was used to analyze normality of data distribution. Two-way ANOVA followed by Tukey post-hoc multiple comparison tests was performed. The normally distributed data were presented as mean ± standard deviation (SD). Significance was set at *p* < 0.05.

## Results

### Early effect of neutralizing IL-1β antibody on maintenance of capillary length in the thalamus is correlated with increased pericyte coverage after diffuse TBI

**Figure 1:**
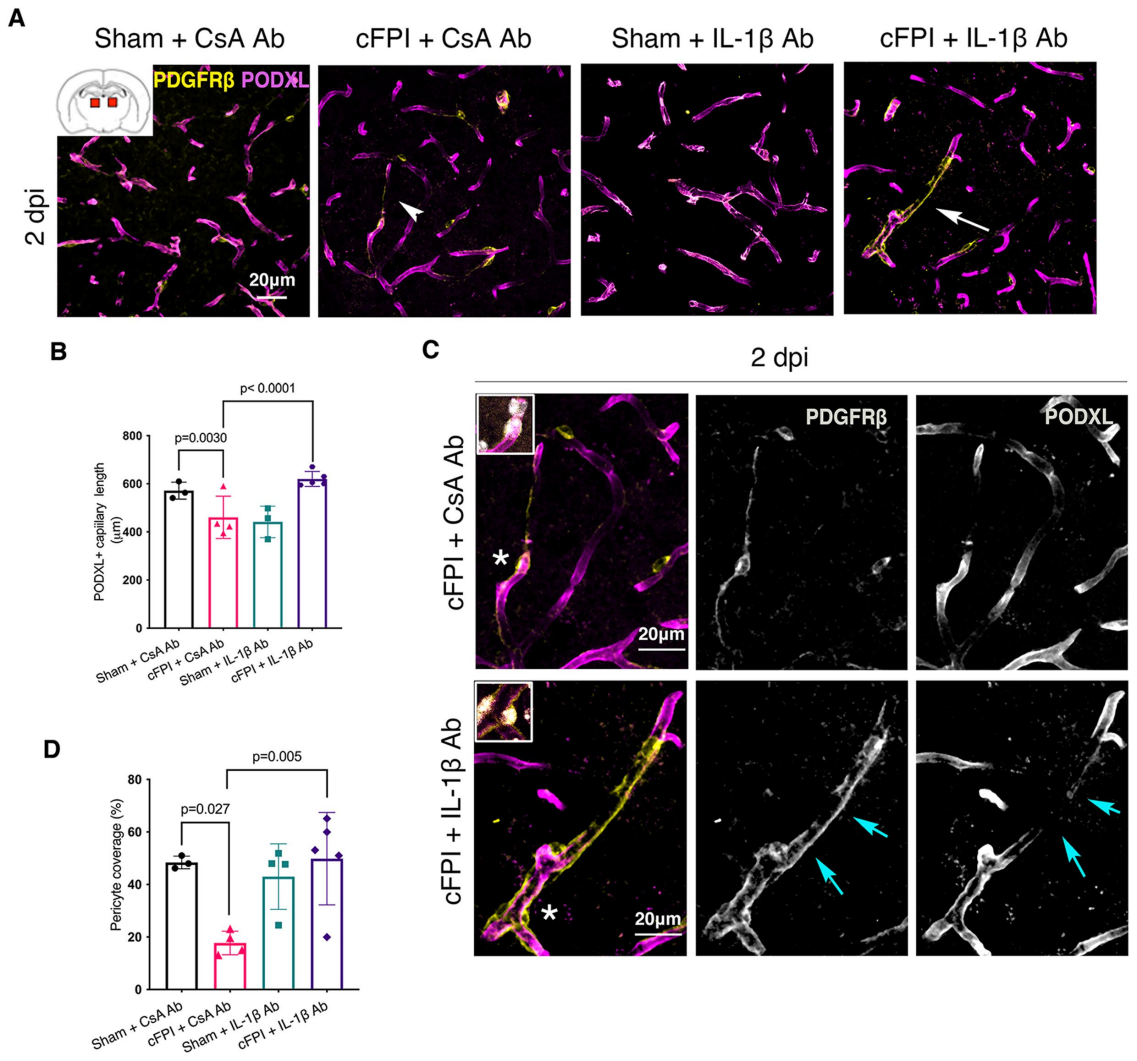
IL-1β neutralization increases pericyte coverage and capillary length in the thalamus at 2 dpi. **(A)** Representative confocal images showing podocalyxin^+^ (PODXL) capillaries (magenta) and PDGFRβ^+^ pericyte coverage (yellow) in the thalamus at 2 dpi. **(B)** Quantification of total capillary length (sham + CsA, *n* = 3; cFPI + CsA, *n* = 4; sham + IL-1β, *n* = 3; cFPI + IL-1β, *n* = 5; mean ± SD) in the cortex at two after the injury. **(C)** High magnification confocal images showing morphology of pericytes in cFPI + CsA Ab (upper panel, white arrow head in **A**) and cFPI + IL-1β Ab group (lower panel, white arrow in **A**). Coverage by PDGFRβ^+^ pericytes in cFPI + IL-1β Ab group, indicated by cyan arrows. Boxed areas show cell nuclei of a pericyte stained with DAPI (grey). **(D)** Quantification of PDGFRβ^+^ pericyte coverage (sham + CsA Ab, *n* = 3; cFPI + CsA Ab, *n* = 4; sham + IL-1β Ab, *n* = 4; cFPI + IL-1β Ab, *n* = 5; mean ± SD).

**Figure 2:**
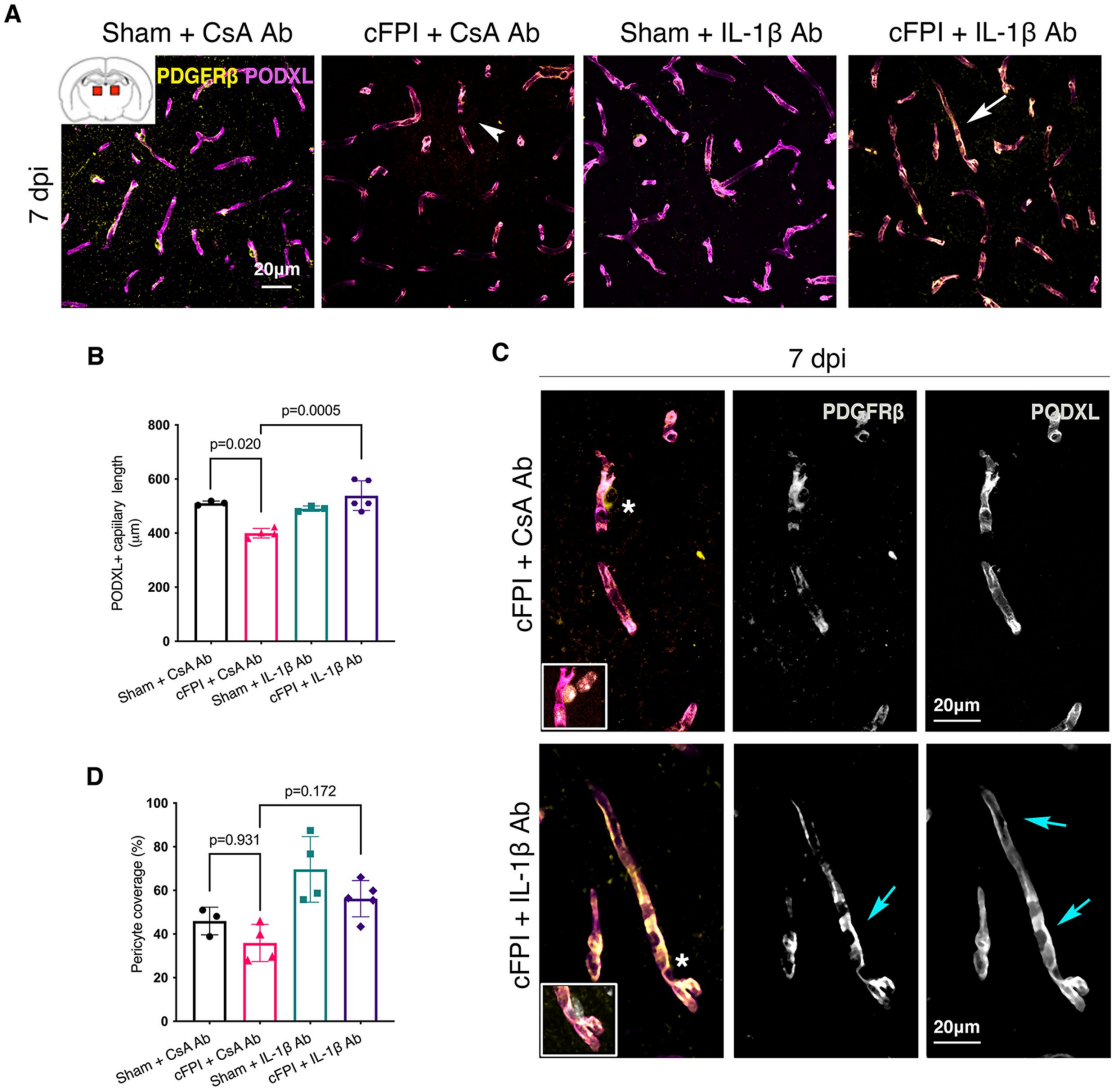
IL-1β neutralization increases only capillary length in the injured thalamus at 7 days post-injury. **(A)** Representative confocal images showing podocalyxin^+^ (PODXL) capillaries (magenta) and PDGFRβ^+^ pericyte coverage (yellow) in the thalamus at 7 dpi. **(B)** Quantification of total capillary length (sham + CsA, *n* = 3; cFPI + CsA, *n* = 4; sham + IL-1β, *n* = 3; cFPI + IL-1β, *n* = 5; mean ± SD) in the thalamus at seven after the injury. **(C)** High magnification confocal images showing morphology of pericytes in cFPI + CsA Ab (upper panel, white arrow head in **A**) and cFPI + IL-1β Ab group (lower panel, white arrow in **A**). Tube-like coverage by PDGFRβ^+^ pericytes in the cFPI + IL-1β Ab group, cyan arrows. Boxed areas show cell nuclei of a pericyte stained with DAPI (grey). **(D)** Quantification of PDGFRβ^+^ pericyte coverage (sham + CsA, *n* = 3; cFPI + CsA, *n* = 4; sham + IL-1β, *n* = 4; cFPI + IL-1β, *n* = 5; mean ± SD).

In the thalamus, cFPI + CsA Ab resulted in a more than 20% decrease in the total capillary length as compared to the sham-injured mice treated with CsA + Ab at 2 and 7 post-injuries (dpi) (2 dpi, *p* = 0.003) (Figures 1A,B); 7 dpi (*p* = 0.020) (Figures 2A,B). This reduction in capillary length was completely normalized by neutralizing anti-IL-1β antibody treatment to levels comparable to those observed in the sham CsA + Ab group both at 2 dpi (*p* < 0.0001) (Figure 1B) and 7 dpi (*p* = 0.0005) (Figure 2B).

**Figure 3:**
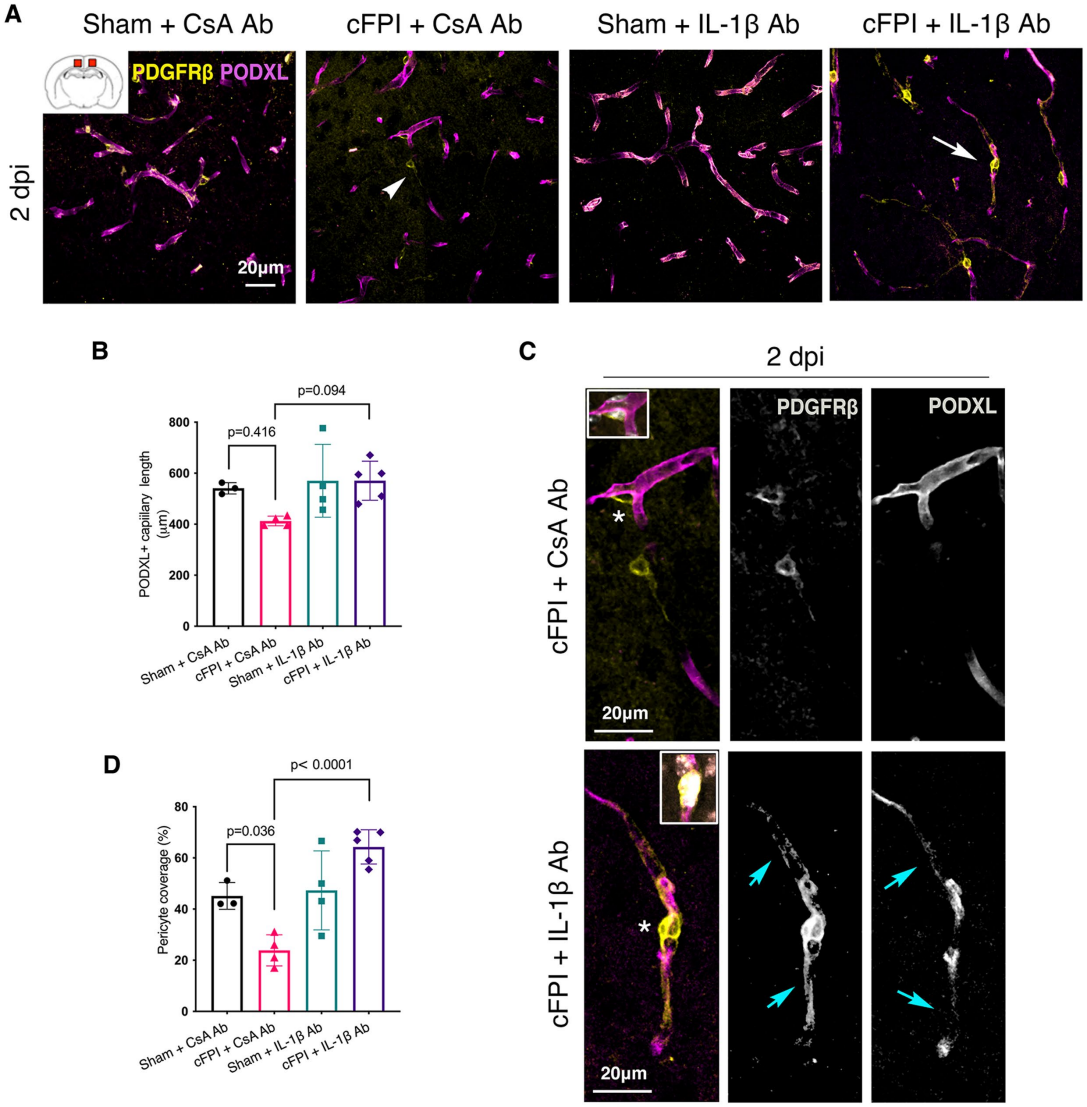
IL-1β neutralization normalizes pericyte coverage in the cortex at 2 dpi. **(A)** Representative confocal images showing podocalyxin^+^ (PODXL) capillaries (magenta) and PDGFRβ^+^ pericyte (yellow) coverage in the cortex at 2 dpi. **(B)** Quantification of total capillary length (sham + CsA Ab, *n* = 3; cFPI + CsA Ab, *n* = 4; sham + IL-1β Ab, *n* = 4; cFPI + IL-1β Ab, *n* = 5; mean ± SD) in the cortex at 2 days after the injury. **(C)** High magnification confocal images showing morphology of pericytes in cFPI + CsA Ab (upper panel, white arrow head in **A**) and cFPI + IL-1β Ab group (lower panel, white arrow in **A**). Higher coverage by PDGFRβ^+^ pericytes in cFPI + IL-1β Ab group, cyan arrows. Boxed areas show cell nuclei of a pericyte stained with DAPI (grey). **(D)** Quantification of PDGFRβ^+^ pericyte coverage (sham + CsA Ab, *n* = 3; cFPI + CsA Ab, *n* = 4; sham + IL-1β Ab, *n* = 4; cFPI + IL-1β Ab, *n* = 5; mean ± SD).

**Figure 4:**
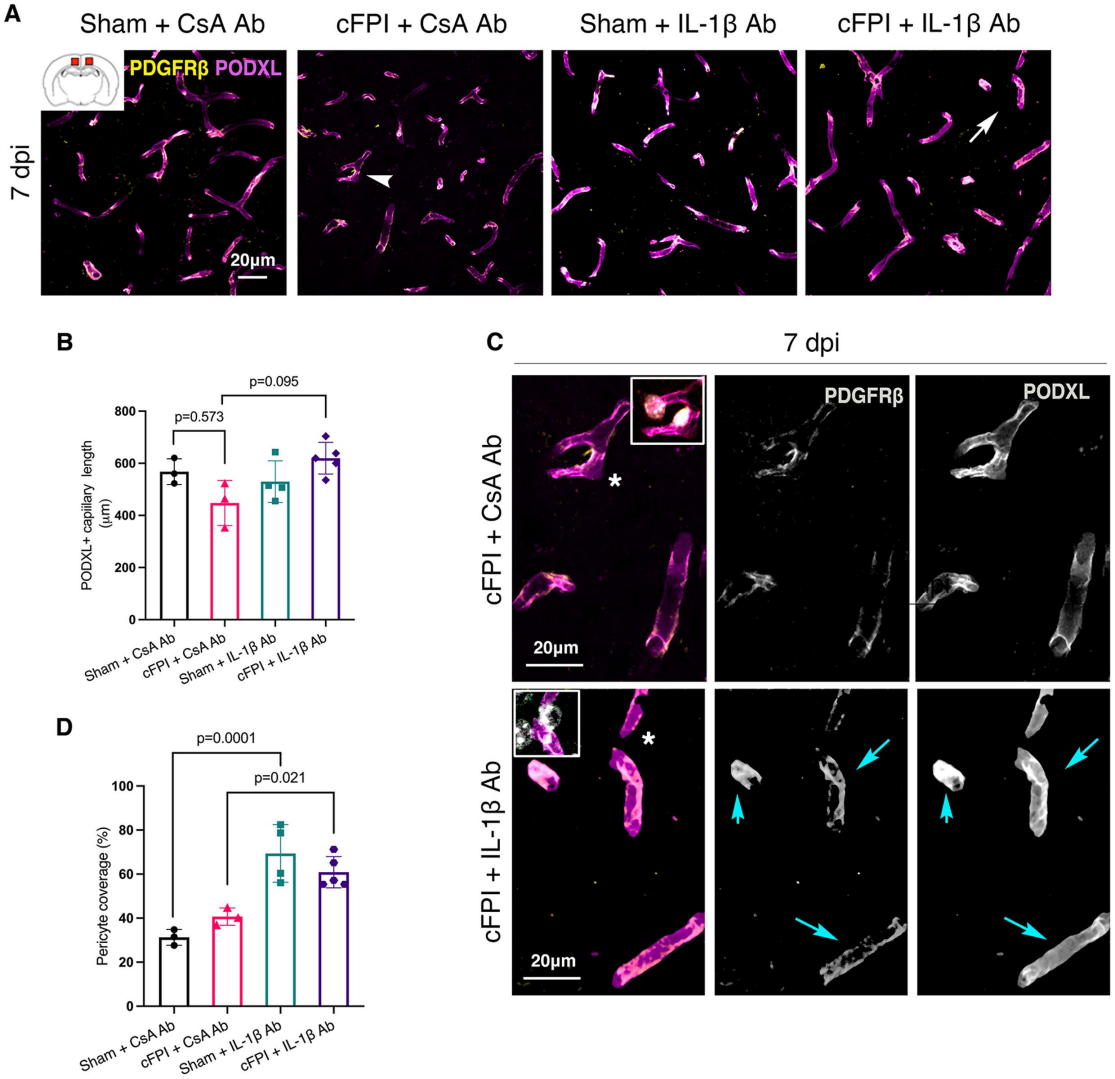
IL-1β neutralization has no significant effect on pericyte coverage and capillary length in the injured cortex at 7 dpi. Representative confocal images showing podocalyxin^+^ (PODXL) capillaries (magenta) and PDGFRβ^+^ pericyte (yellow) coverage in the cortex at 7 dpi. (**B**) Quantification of total capillary length; (sham + CsA Ab, *n* = 3; cFPI + CsA Ab, *n* = 3; sham + IL-1β Ab, *n* = 4; cFPI + IL-1β Ab, *n* = 5; mean ± SD) in the cortex at 7 days post- injury. (**C**) High magnification confocal images showing morphology of pericytes in cFPI + CsA Ab (upper panel, white arrow head in **A**) and cFPI + IL-1β Ab group (lower panel, white arrow in **A**). PDGFRβ^+^ pericytes coverage in cFPI + IL-1β Ab group, cyan arrows. Boxed areas show pericyte cell nuclei, stained with DAPI (grey). (**D**) Quantification of PDGFRβ^+^ pericyte coverage (sham + CsA Ab, *n* = 3; cFPI + CsA Ab, *n* = 3; sham + IL-1β Ab, *n* = 4; cFPI + IL-1β Ab, *n* = 5; mean ± SD).

To determine changes in pericyte coverage, we examined pericytes processes in relation to endothelial cells forming the microcapillary wall (Figures 1–4). PDGFRβ^+^ pericytes were identified by their prominent cell body and processes, and their perivascular location adjacent to PODXL expressing blood vessels was confirmed. We observed varying degrees of pericyte coverage in response to the cFPI injury and neutralizing antibody treatments (Figures 1–4). There was loss of PODXL^+^ expression in capillaries in areas where pericytes coverage was completely or partially absent in the thalamus of cFPI mice receiving control CsA Ab treatment (Figures 1, 2). At 2 dpi, cFPI caused ca 65% decrease (*p* = 0.027) in PDGFRβ^+^ pericytes coverage in the thalamus (Figures 1A,D). The cFPI group treated with neutralizing IL-1β antibody, however, reached sham control values and had significantly higher pericyte coverage than the cFPI + CsA Ab control group (*p* = 0.005) (Figures 1A,C,D). Unlike at 2 dpi, cFPI did not cause significant changes in coverage of PDGFRβ^+^ pericytes when compared to sham-operated mice at 7 dpi in the thalamus (Figures 2A,C,D).

### Neutralization of IL-1β results in higher pericyte coverage in the cortex after diffuse TBI

In the cortex, there was no significant difference in the capillary length at 2 dpi between groups (sham + CsA Ab and cFPI + CsA Ab) (*p* = 0.416); CsA Ab and cFPI + IL-1β Ab (*p* = 0.094) (Figures 3A,B). In the brain injured group, loss of PODXL^+^ expression in capillaries was found in areas where pericytes coverage was completely or partially absent (Figure 3C, upper panels). While there was no reduction in capillary length, the cFPI resulted in a 50% decrease (*p* = 0.036) in pericyte coverage when compared to the sham group at 2 dpi (Figure 3D). Pericyte coverage was 47% higher in neutralizing IL-1β antibody antibody-treated mice then in the injured group that received only control antibody (CsA Ab) at 2 dpi (*p* < 0.0001) (Figures 3C,D).

At 7 dpi, the total capillary length in the cFPI + CsA Ab group was similar to the sham-injured mice treated with CsA Ab (*p* = 0.573) (Figure 4A). Although the capillary length of cFPI + IL-1β Ab-treated mice was higher and comparable to sham-values, it did not reach statistical significance when compared to the cFPI + CsA Ab group (*p* = 0.095) (Figures 4A–C). However, the brain injured mice that received neutralizing IL-1β antibody had significantly higher pericyte coverage than cFPI + CsA Ab group (*p* = 0.021) (Figures 4C,D), whereas pericyte coverage was not altered in cFPI + CsA Ab mice compared to the sham-injured CsA Ab group at 7 dpi (Figures 4A,D). There was significant difference between sham + CsA Ab and sham + IL-1β groups (*p* = 0.002) (Figure 4D).

### Neutralization of IL-1β does not affect the number of PDGFRβ expressing pericytes after diffuse TBI

**Figure 5:**
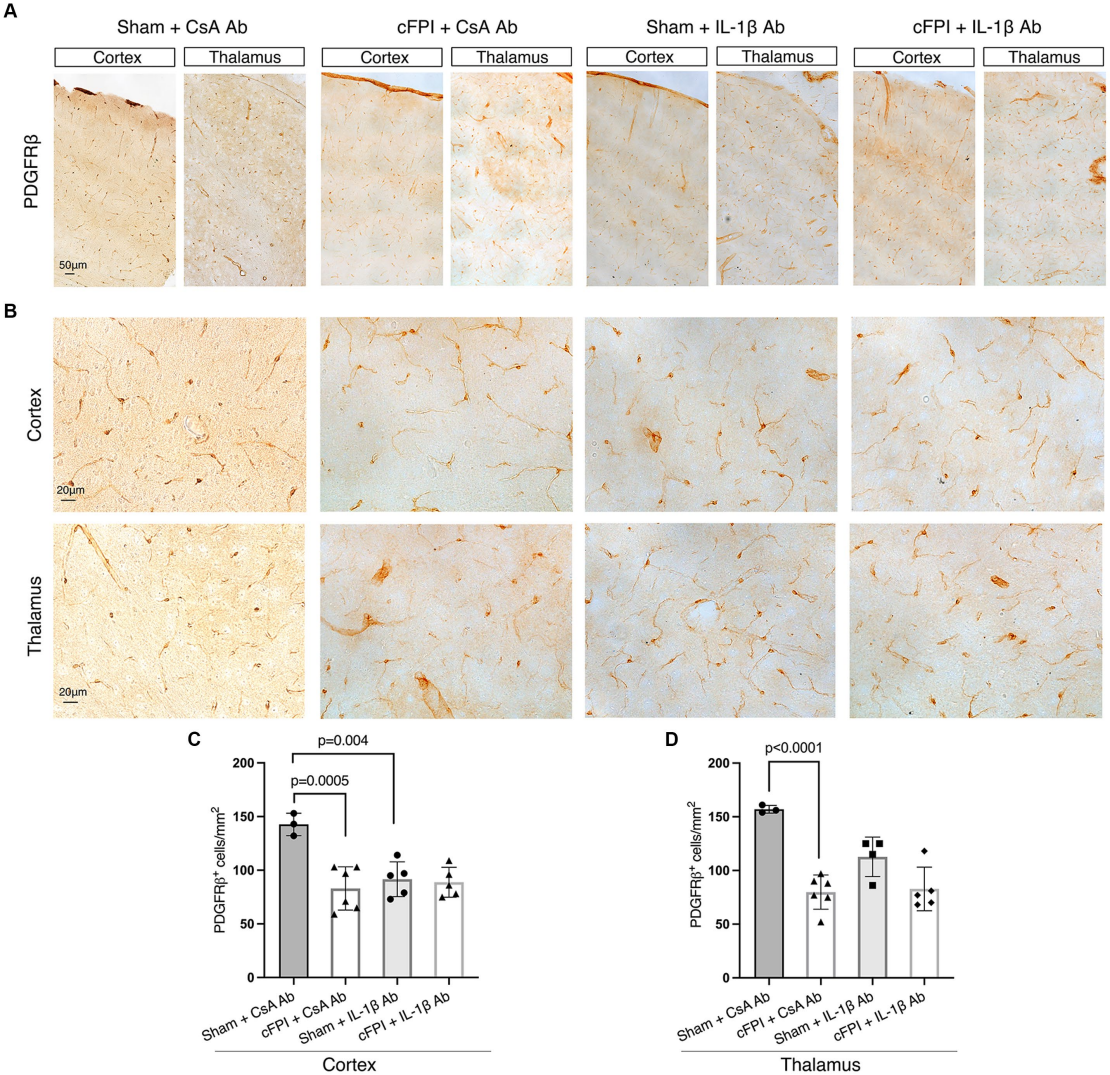
IL-1β neutralization does not influence PDGFRβ^+^ pericyte numbers in the cortex and thalamus at 2 days post-injury. **(A,B)** Representative images showing PDGFRβ staining in the cortex and thalamus at 2 dpi **(A)**, in higher magnification in **(B)**. (**C,D**) Quantification of the number of PDGFRβ^+^ pericytes in cFPI + IL-1β Ab vs. other groups (sham + CsA Ab, sham + IL-1β Ab, and cFPI + CsA Ab) in the cortex **(C)** and thalamus **(D)**. (**C**) Neutralizing IL-1β had no effect on the number of PDGFRβ^+^ pericytes in the cortex at 2 dpi (sham + CsA Ab, *n* = 3; cFPI + CsA Ab, *n* = 6; sham + IL-1β Ab, *n* = 5; cFPI + IL-1β Ab, *n* = 5). (**D**) In the thalamus, the number of PDGFRβ^+^ pericytes in the cFPI + CsA Ab animals was not influenced by IL-1β neutralizing antibody treatment (sham + CsA Ab, *n* = 3; cFPI + CsA Ab, *n* = 6; sham + IL-1β Ab, *n* = 4; cFPI + IL-1β Ab, *n* = 5; mean ± SD).

**Figure 6:**
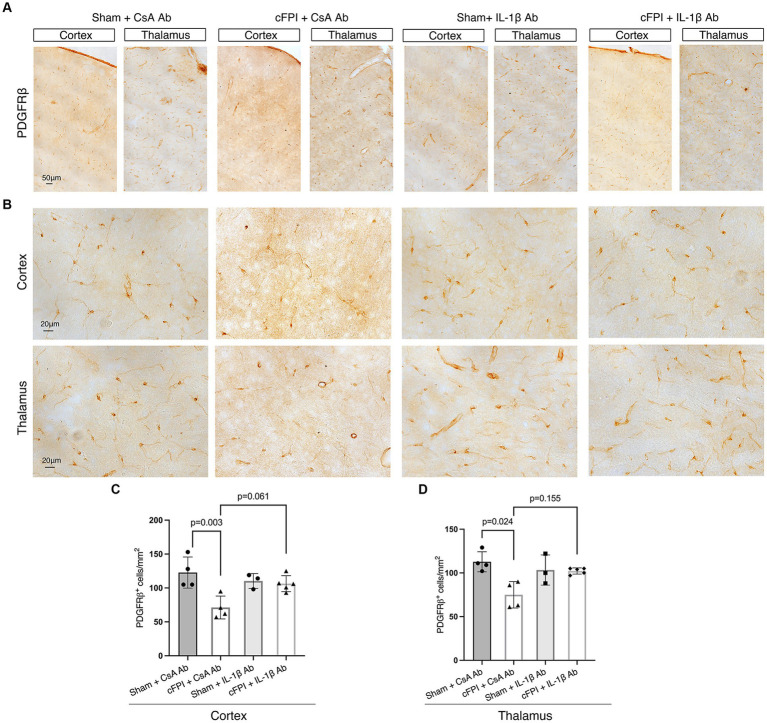
The number of PDGFRβ^+^ pericytes remains unchanged in the cortex and thalamus after IL-1β neutralization at 7 days post-injury. (**A,B**) Representative images showing PDGFRβ staining in the cortex and, thalamus at 7 dpi (**A**), higher magnification in (**B**). (**C,D**) Quantification of the number of PDGFRβ^+^ pericytes in cFPI + IL-1β Ab animals compared with other groups (sham + CsA Ab, sham + IL-1β Ab, and cFPI + CsA Ab) in the cortex **(C)** and thalamus **(D)**. (**C**) At 7 dpi, neutralizing anti-IL-1β had no effect on the number of PDGFRβ^+^ pericytes in the cortex (sham + CsA Ab, *n* = 4; cFPI + CsA Ab, *n* = 4; sham + IL-1β Ab, *n* = 3; cFPI + IL-1β Ab, *n* = 5; mean ± SD) and thalamus (sham + CsA Ab, *n* = 4; cFPI + CsA Ab, *n* = 4; sham + IL-1β Ab, *n* = 3; cFPI + IL-1β Ab, *n* = 5; mean ± SD).

We finally examined cFPI-induced changes in pericytes distribution in the cortex and thalamus of all groups using brightfield microscopy and image analyses for PDGFRβ, a marker for brain pericytes. PDGFRβ^+^ pericytes with a prominent cell body and long process were identified throughout the cortex and thalamus (Figures 5A,B), distributed in different cortical layers and thalamic regions in all groups at 2 dpi (Figure 5) and 7 dpi and (Figure 6). At 2 dpi, quantitative analysis showed a 50% decrease in the numbers of PDGFRβ^+^ pericytes in the cortex (*p* = 0.0005) (Figure 5C) and thalamus (*p* < 0.001) (Figure 5D) in the cFPI + CsA Ab group when compared to the sham + CsA Ab group, indicating that cFPI leads to pericyte loss. However, pericyte numbers in the cFPI + IL-1β Ab and sham + IL-1β Ab groups remained unchanged at 2 dpi.

Similarly, at 7 dpi, the number of PDGFRβ^+^ pericytes was significantly decreased in the cortex (*p* = 0.003) and thalamus (*p* = 0.024) in the cFPI + CsA Ab group when compared to the sham + CsA Ab group illustrating that the pericyte loss was persistent in the cFPI + CsA Ab group (Figures 6A–D). At 7 dpi, pericytes numbers were not significantly increased by neutralizing anti-IL-1β antibody treatment in any of the groups compared to the cFPI + CsA Ab group (*p* = 0.061 cortex) (Figure 6C); (*p* = 0.155 thalamus) (Figure 6D).

## Discussion

In experimental and human TBI, the production of IL-1β increases rapidly after the injury and this may be directly involved in neuroinflammatory processes associated with neurodegeneration (11, 17, 37). Here, we demonstrated that IL-1β neutralization has an impact on preservation of capillaries and especially increases pericyte coverage, and those effects were more pronounced in the thalamus, a region particularly sensitive to the TBI in this model.

Diffuse axonal and diffuse vascular injuries are the most common hallmarks of TBI, where widespread lesions are commonly observed throughout white and deep grey matter structures. The cFPI model is clinically relevant experimental rodent model to examine its pathology in brain regions not directly targeted by the physical impact of the delivered fluid pressure pulse (34, 37, 39). The cFPI model results in widespread pathology, also in brain regions not directly targeted by the physical impact. The thalamus is a critical brain region relaying sensory information to the cortex. It also involves several other vital functions, such as cognition, motor control, and sleep-wake cycle regulation (40). The thalamus is particularly vulnerable to the effects of diffuse injury in the experimental TBI, and this has significant clinical implications (7, 8). For instance, long-term MRI imaging studies have revealed a reduction in cerebral flow in the thalamus of individuals with mild TBI, which was associated with cognitive impairment (21). In addition to axonal injury and inflammation, interconnected capillary network in remote areas can be at risk of disruption by mechanical forces of a diffuse brain injury. In this study, we showed that cFPI resulted in a significant decrease in total capillary length in the thalamus 2 and 7 dpi, findings consistent with previous work demonstrating that cFPI caused a significant decrease in microvascular density (25), which was reversed by neutralization of IL-1β. The reduction in vascular density during acute phases of lateral fluid percussion models in rats has been shown to correlate with functional decrements (23, 25).

The central fluid percussion resulted in a decreased number of PDGFRβ-expressing pericytes in the thalamus and cortex at 2 and 7 days after TBI. These findings are supported by previous studies in rodent FPI models where regional pericyte loss is observed (41). Given that pericytes are highly susceptible to brain injury and ischemia, any disruption of the crosstalk between pericyte and the vascular wall can lead to pericyte malfunction and degeneration during the subacute phase of brain injury (29, 30, 41). In the acute phase of TBI, a subgroup of pericytes migrates away from the blood vessels while the remaining ones around the endothelium undergo degeneration and display apoptotic or necrotic cellular changes (42, 43). However, the anti-IL-1β neutralizing antibody did not influence the number of pericytes at any time point arguing against a role for IL-1β in TBI-induced pericyte death in the cortex and thalamus.

The heterogeneity in the morphological pattern of pericyte processes has been described in several studies (44, 45), indicating the complexity of their structure and function. Several studies using high-resolution optical imaging have attempted to classify capillary pericytes based on their morphology in recent years. There are two main types of capillary pericytes—“thin-strand pericytes” and “mesh pericytes” (46). Thin strand pericytes display a prominent cell body and elongated processes, while mesh pericytes have higher levels of vessel coverage. These findings suggest that classifying capillary pericytes based on their morphology may provide valuable insights into their function and help understand their role in various pathological processes. It is important to note that TBI can result in increased blood-brain barrier permeability, leading to the dysfunction of cerebral autoregulation and edema formation (9, 20, 22). Therefore, our current study shows that pericyte remodeling is crucial to maintaining capillary network function following diffuse brain injury. The cFPI resulted in a rapid decrease in PDGFRβ^+^ pericyte coverage in thalamus and the cortex at 2 days post injury, however, these changes were no longer detectable in both regions at 7 days post injury. Regardless of structural heterogeneity, we observed that of PDGFRβ^+^ pericytes extended their process into the uncovered capillary bed in vessel areas where PODXL expression was absent at 2 dpi in both regions. This may reflect their early contribution to neovascularization and vascular remodeling after brain injury (26, 47). On the other hand, as pericytes regulate the stabilization and the function of blood vessels, it is likely that they extend their processes to preserve their endothelial contact after pericyte loss (26, 48). Most importantly, neutralizing IL-1β antibody treatment after diffuse TBI increased pericyte coverage in the thalamus 2 days after injury, which aligns with our previous findings that neutralizing IL-1β antibody reduced TBI-induced hemispheric edema at 2 dpi (20).

Chronic follow-up studies using lateral FPI model in rats show that long-lasting angiogenic response in the thalamus showed difference compared to cortical areas after the injury, which was associated with functional abnormalities (49). Although potential mechanisms underlying the mechanisms of vascular modulation require further investigation, the coinciding presence of inflammation with capillary reduction after TBI indicates that a post-injury inflammatory response may be involved in modulation of vascular response in a time/region dependent way. Here, we found that brain pericytes, highly expressing PDGFRβ, acquired more rounded morphology around the capillaries in the thalamus and cortex at 7 dpi. These pericytes with full coverage around the precapillary exhibit the highest capability of altering the cerebrovascular flow resistance, particularly in the thalamus (26). Consequently, they may be responsible for regulating blood flow through the capillaries while protecting the downstream capillary bed and the thalamus from any unfavorable pressure fluctuations (18). Indeed, the thalamus is highly vascularized by arteries maintaining its normal functioning, and any disruption in their blood supply can lead to significant neurological deficits, including cognitive impairment, and motor deficits (5, 21, 50). Our previous study used the multivariate concentric square field (MCSF) and the Morris water maze (MWM) tests to assess functional outcomes post-injury. We showed that treatment with the IL-1β-neutralizing antibody, as used in the present study, attenuated stereotypic behaviors induced by TBI at 2 dpi and 9 dpi (35). However, these tests might not detect thalamic dysfunction after diffuse TBI. Advanced imaging techniques including MRI may be necessary to accurately assess changes in the blood flow and its relation to neurological functions and inflammation of the thalamus following TBI. Nevertheless, acute and chronic inflammation in the different brain regions after vascular damage may lead to behavioral changes even in the absence of detectable neuronal pathology (50). Using lateral FPI in rats, acute and persistent inflammation in the brain after vascular damage leads to neurobehavioral changes that could be reversed using immunotherapy (51). The primary effect IL-1β on endothelial cells in the brain is to increase vascular permeability and inflammation in both trauma-induced and ischemic induced lesions (52, 53); however, our findings show that neutralizing IL-1β antibody may have region-dependent diverse functions on the cerebral vasculature. Furthermore, the administration of IL-1β neutralizing antibody increased pericyte coverage in sham-injured mice similar to injured cortex at 7 dpi, indicating that the surgical procedure used also in sham injury could trigger a minor delayed inflammatory response in the cortex.

There are several limitations of our study to consider. Although previous studies using experimental mouse models reported evidence of thalamic injury and neuronal death following TBI (54, 55), in the present study we did not analyze the possible effect of neutralizing IL-1β antibody on neuronal loss in the thalamus and cortex. However, in previous studies in mice, this treatment strategy was found to reduce the TBI-induced loss of cortical tissue indicating a potential effect on neuronal survival (20). The antibody is known to reach the mouse brain in therapeutic concentrations (>30 μg/g brain tissue) at both 24 and 72 h post-injury. Although the dosing interval for neutralizing IL-1β antibody was similar to that of previous studies (35), we did not specifically determine the endogenous levels of IL-1β in the cortex and thalamus as well as in the vascular cells after the cFPI and antibody treatment in the present study. However, the increase of IL-1β is expected to occur in the first post-injury hours (15) and at time of our study endpoints at 2 and 7 dpi, tissue IL-1β levels are presumably low (56, 57). In addition, it remains to be investigated in future studies whether neutralization of IL-1β antibody may influence blood flow regulation, capillary diameter, and BBB integrity.

Overall, our study raises awareness that the modulation of the damaged microvascular environment is an important therapeutic intervention in TBI, in which capillary regression is a factor.

## Data availability statement

The original contributions presented in the study are included in the article/supplementary material, further inquiries can be directed to the corresponding author.

## Ethics statement

All experiments were approved by the Uppsala County Animal Ethics board and followed the regulations of the Swedish Animal Welfare Agency. The study was conducted in accordance with the local legislation and institutional requirements.

## Author contributions

IÖ: Conceptualization, Formal analysis, Investigation, Methodology, Software, Validation, Visualization, Writing – original draft. FC: Investigation, Methodology, Writing – original draft. JF: Methodology, Writing – review & editing. NM: Conceptualization, Funding acquisition, Project administration, Resources, Supervision, Writing – review & editing. GP: Conceptualization, Funding acquisition, Project administration, Resources, Supervision, Writing – review & editing.
